# A Clinical Lecture on Goitre

**Published:** 1898-06-04

**Authors:** J. Lacy Firth

**Affiliations:** AssistantSurgeon to the Bristol General Hospital


### Medical Progress and Hospital Clinics.

[*The Editor will be glad to receive offers of co-operation and contributions from members of the profession. All letters should be addressed to* The Editor, at the Office, 28 & 29, Southampton Street, Strand, London, W.C.]

### A CLINICAL LECTURE ON GOITRE.

By J. Lacy Firth, M.D., M.S., F.R.C.S., Assistant-Surgeon to the Bristol General Hospital.

(*Continued from page* 146.)

It is most frequently the bilateral parenchymatous enlargements which give rise to serious dyspnœa. There is one obvious cause of this dyspnœa, viz., pressure on the trachea. The trachea is usually diminished in calibre by the increased pressure, and its shape altered. Always in the general enlargements the sides of the air tube are pressed inwards. The lumen may thus be reduced to a mere antero-posterior slit, the so-called scabbard trachea. Sometimes a portion of the thyroid will enlarge behind the sternum, and compress the trachea from before backwards, but this is rare. It is obvious that the narrowing of the trachea, though usually found in these cases, does not completely explain the origin of the sudden attacks of dyspnœa. Many theories have been advanced to explain these attacks and their sudden onset, but none of them are very satisfactory. In some cases the enlarged thyroid, or a cyst in it, suddenly becomes the seat of hæmorrhage, and the increased pressure reduces the lumen of the trachea to such a small size that urgent dyspnœa results. In some cases, though the condition seems to be rare, the cartilaginous rings are much softened, and the dyspnœa, it is suggested, may be due to kinking of the softened trachea by an alteration in the position of the neck on falling asleep, or at other times. Pressure of the swelling upon the recurrent laryngeal nerves leading to abductor paralysis or spasm of the glottis does not explain these cases, for tracheotomy above the isthmus does not relieve the dyspnœa. Dr. Hurry, of Reading, thinks that the dyspnœa once started tends to increase, because the contraction of the extraordinary muscles of respiration press upon the thyroid, and so upon the trachea, still further narrowing its lumen. The dyspnœa, as it increases, may then cause more and more forcible contraction of these muscles, and more and more narrowing of the trachea, a vicious circle being established. This theory certainly yields an intelligible explanation of the attacks due to an exciting cause which starts the vicious cycle of events, *e.g.*, fright; and it is possible that in those cases where no exciting cause of such a kind has been detected, that one was present nevertheless, and that it was slight and therefore unobserved. It is interesting that dyspnœa was found to be a characteristic symptom of athyroidism produced experimentally in animals by Mr. Horsley. How are these attacks to be relieved? Tracheotomy or laryngotomy is not sufficient. In addition a tube, for instance, a catheter with a terminal opening, must be passed down the trachea to a point beyond the lower level of the compressed portion. Excision of the thyroid isthmus has been tried in some cases and found to be quite unreliable. It is not the isthmus which presses upon the trachea, but the lateral lobes. The shape of the compressed trachea shows that. When the isthmus has been removed in some of these cases the lateral lobes have come together in the middle line, as though the isthmus had previously wedged them apart; and when the trachea has also been opened, and a catheter passed beyond the obstruction, that instrument has had to be left in for many days, even three weeks, all attempts to breathe without it resulting in a recurrence of the dyspnœa. Ultimately the excision of the isthmus usually produces a decrease in the size of the lateral lobes, and it is not until this result has followed the operation that the tracheal pressure and the dyspnœa are effectually relieved. Dysphagia is a symptom in some cases of goitre, but is not nearly so common as dyspnœa. It is the result, of course, of pressure upon the œsophagus. Venous congestion of the head and neck may result in a similar way from pressure in the veins of the neck, and giddiness often accompanies it.

Before leaving the symptoms met with in cases of goitre, I must refer to the condition known as sporadic cretinism, because the condition is sometimes met with in association with a large thyroid, though usually the gland is undeveloped. At birth or in infancy, the victims of this disease develop symptoms very similar to those of myxœdema in adults, and, in addition, their growth is imperfect physically and intellectually. The patients are dwarfs, and, however long they live, retain a childlike appearance in many respects. The close resemblance of the signs and symptoms of cretinism to those of myxœdema, and the similarity of both to the condition which has been produced in many persons accidentally, and in animals experimentally, by the complete removal of the thyroid gland (cachexia strumipriva), and the further fact that all three conditions are ameliorated to a marked degree by the ingestion of thyroid gland extract, show that they are due to an imperfection in either the quantity or quality or in both of the "internal secretion" of the gland. That cretins exist with large thyroids shows that the secretion of an enlarged gland may be as imperfect in quality or quantity as that of an atrophied gland, such as is met with in myxœdema. It is remarkable that the administration of thyroid extract in some of these cases of cretinism with large thyroids, the giving of an agent of nutrition which the gland itself is failing to provide, will very much diminish the size of the enlarged gland. It is easier to understand how thyroid extract will produce atrophy in a normal thyroid gland than how it will cause atrophy in a hypertrophied gland, which is already in important respects functionless.

The treatment of goitres depends largely upon the urgency of the associated symptoms. In a large proportion of cases there are few, if any, symptoms clearly due to the state of the gland. Some enlargement of the gland, for example, is common in anæmic girls without special symptoms. The proper treatment for such cases is, in the first place, to secure good hygienic surroundings. Fresh air and sunlight are required, and these can often be most advantageously obtained by residence in fairly high altitudes. The anæmia must be treated appropriately with iron, arsenic, laxatives, and good food. In addition, iodine should be used, as it appears often to exercise a very beneficial effect upon the enlarged gland. It should be given internally in the form of iodides in large doses, and applied locally over the gland by the inunction of the compound iodine ointment or that of iodide of lead or one of biniodide of mercury. As the health improves the thyroid often diminishes in size, or at least ceases to increase, and though it often remains permanently larger than natural it gives no further trouble. In all cases then of simple or adenomatous goitre in which the symptoms are not so urgent as to demand an immediate reduction in the size of the swelling, the iodide treatment should be tried, and you will find in practice that the majority of cases met with will fall into this group. In another group of cases advice may be sought because the swelling is unsightly, or because it is a source of discomfort by reason of its size and weight. If in such cases the iodine treatment fails after trial to give the desired relief, or if at the outset it is obvious that it will fail, as would be the case, for example, if the enlargement were due to a large cyst, then operative treatment will claim consideration.

(*To be continued.*)

